# Comparison of Neutralizing Activity between Vaccinated and Unvaccinated Hospitalized COVID-19 Patients Infected with Delta, Omicron BA.1, or Omicron BA.2 Variant

**DOI:** 10.3390/microorganisms12030509

**Published:** 2024-03-02

**Authors:** Keun Ju Kim, Seo-Jin Park, Seung Gyu Yun, Sang Wook Kim, Myung-Hyun Nam, Eun Kyong Shin, Eun-Ah Chang, Dae Won Park, Chang Kyu Lee, Young Kyung Yoon, Yunjung Cho

**Affiliations:** 1 Department of Laboratory Medicine, Korea University Anam Hospital, Korea University College of Medicine, Seoul 02841, Republic of Korea; themicrobialworld@gmail.com (K.J.K.); koryun@korea.ac.kr (S.G.Y.); kkshws@naver.com (S.W.K.); yuret@korea.ac.kr (M.-H.N.); cklee5381@gmail.com (C.K.L.); 2 Department of Laboratory Medicine, Ajou University School of Medicine, Suwon 16499, Republic of Korea; seojinpark@hotmail.com; 3 Department of Sociology, Korea University, Seoul 02841, Republic of Korea; eunshin@korea.ac.kr; 4 Department of Laboratory Medicine, Korea University Ansan Hospital, Korea University College of Medicine, Ansan 15355, Republic of Korea; eunah_chang@scllab.co.kr; 5 Department of Laboratory Medicine, Seoul Clinical Laboratories, Yongin-si 16954, Republic of Korea; 6 Division of Infectious Diseases, Department of Internal Medicine, Korea University Ansan Hospital, Korea University College of Medicine, Ansan 15355, Republic of Korea; pugae1@korea.ac.kr; 7 Division of Infectious Diseases, Department of Internal Medicine, Korea University Anam Hospital, Korea University College of Medicine, Seoul 02841, Republic of Korea

**Keywords:** COVID-2019, surrogate virus neutralization test, SARS-CoV-2 variants, vaccination, cross-neutralizing activity

## Abstract

Background: Understanding the immune response to evolving viral strains is crucial for evidence-informed public health strategies. The main objective of this study is to assess the influence of vaccination on the neutralizing activity of SARS-CoV-2 delta and omicron infection against various SARS-CoV-2 variants. Methods: A total of 97 laboratory-confirmed COVID-19 cases were included. To assess the influence of vaccination on neutralizing activity, we measured the neutralizing activity of SARS-CoV-2 delta or omicron (BA.1 or BA.2) infection against wild-type (WT), delta, BA.1, and BA.2, with the results stratified based on vaccination status. Results: The neutralizing activity against the WT, delta, and omicron variants (BA.1 and BA.2) was significantly higher in the vaccinated patients than those in the unvaccinated patients. In the unvaccinated individuals infected with the delta variant, the decrease in binding to BA.1 and BA.2 was statistically significant (3.9- and 2.7-fold, respectively) compared to the binding to delta. In contrast, vaccination followed by delta breakthrough infection improved the cross-neutralizing activity against omicron variants, with only 1.3- and 1.2-fold decreases in BA.1 and BA.2, respectively. Vaccination followed by infection improved cross-neutralizing activity against WT, delta, and BA.2 variants in patients infected with the BA.1 variant, compared to that in unvaccinated patients. Conclusions: Vaccination followed by delta or BA.1 infection is associated with improved cross-neutralizing activity against different SARS-CoV-2 variants. The enhanced protection provided by breakthrough infections could have practical implications for optimizing vaccination strategies.

## 1. Introduction

The emergence of the novel coronavirus disease 2019 (COVID-19) caused by the severe acute respiratory syndrome coronavirus 2 (SARS-CoV-2) has posed an unprecedented global challenge to public health [1]. Since its initial outbreak, the virus has continuously evolved, giving rise to a myriad of variants with distinct genetic profiles [2]. These variants, often characterized by changes in key spike protein regions, have raised concerns about their potential to impact the pandemic course, including vaccine efficacy and disease severity [3]. The virus can propagate more quickly and efficiently because of all those variations combined with other previously observed ones result in mutations, primarily in the spike protein gene [4]. The protective antiviral immunity developed following infection or vaccination is weakened by the global emergence of new SARS-CoV-2 variants [5]. Numerous elements of COVID-19 immunity have been outlined, but there persists a gap between the anticipated understanding and the explanations provided, especially concerning the evolution of SARS-CoV-2 and the emergence of novel variants [1,5]. The emergence of novel SARS-CoV-2 variants, such as delta, omicron, and subsequent lineages, has underscored the need to assess immune responses induced by natural infection or vaccination followed by infection against these evolving variants [6]. The presence of neutralizing antibodies is associated with protection against COVID-19 infection in both infected and diseased individuals [7]. Therefore, it is critical to understand the extent and effectiveness of neutralizing activity against SARS-CoV-2 post-vaccination in a variant-specific manner to guide vaccination policies and pandemic preparedness efforts [8,9]. Previous studies have examined neutralizing immunity in breakthrough infections resulting from delta and omicron, comparing them to infection-naive vaccinated individuals, revealing a robust response post-breakthrough infection [9,10,11]. However, the development of neutralizing antibody responses in delta and omicron breakthrough infections, especially when compared to unvaccinated individuals, remains largely unexplored, due to the limited number of unvaccinated individuals in many countries [12]. Here, we evaluated the neutralizing activity of delta and omicron infection against different SARS-CoV-2 lineages between unvaccinated and vaccinated patients.

## 2. Materials and Methods

### 2.1. Study Participants

This retrospective cohort study included 97 patients with laboratory-confirmed COVID-19 who were hospitalized at the Korea University Anam Hospital between November 2021 and March 2022. Since we designed to determine SARS-CoV-2 variants and neutralizing activity, patients with remnant respiratory and serum samples were included.

### 2.2. Data Sources

The clinical data were collected from patient electronic medical records.

#### 2.2.1. Baseline Characteristics

Baseline characteristics, including the time from symptom onset to hospital admission, age, sex, nationality, comorbidities, immunosuppression, vaccination status, and the cycle threshold (Ct) value of the first PCR on admission, were extracted from the electronic medical records of the Korea University Anam Hospital. The COVID-19 vaccination status was defined as the number of COVID-19 inoculations available in South Korea, including BNT162b2 (BNT; Pfizer-BioNTech), mRNA-1273 (mRNA; Moderna), ChAdOx1 nCoV-19 (ChAd; AstraZeneca), and NVX-CoV2373 (Novavax). “Vaccinated” can be defined as the completion of two-dose homologous (BNT-BNT or ChAd-ChAd) or heterologous (ChAd-BNT) primary schedules, along with two weeks thereafter, as well as the administration of a third COVID-19 vaccine booster dose (BNT-BNT-BNT, ChAd-BNT-BNT, ChAd-ChAd-BNT, ChAd-ChAd-mRNA). One partially vaccinated patient received a single COVID-19 vaccine dose (NVX-CoV2373). Ancestral-virus monovalent vaccines were administered, not variant-adapted vaccines. Unvaccinated patients had not received any COVID-19 vaccine doses. Comorbidities were identified as described in a previous study [13] and expressed as Charlson Comorbidity Index (CCI) scores (categorical; 0, 1, and ≥2).

#### 2.2.2. Variant Identification

The spike gene target status in the PCR is used to identify each variant. We used the PowerChek SARS-CoV-2 S-gene mutation detection kit (Ver3.0) (Kogene Biotech, Seoul, Republic of Korea) to determine the variant type using SARS-CoV-2-positive remnant respiratory samples from all patients.

#### 2.2.3. Surrogate Virus Neutralization Test

The cPass SARS-CoV-2 surrogate virus neutralization test (sVNT) (GenScript Biotech, Piscataway, NJ, USA) was used to measure the neutralizing activity of patient serum samples against SARS-CoV-2 variants. The sVNT utilizes the recombinant receptor-binding domain (RBD) of the SARS-CoV-2 spike protein to detect antibodies that block the RBD from binding to the hACE2 receptor. The recombinant RBD from wild-type (WT), delta, and omicron (BA.1 and BA.2) variants and recombinant human ACE2-coated 96-well plates were used as previously described [14]. Samples with ≥30% inhibition were considered positive for SARS-CoV-2-neutralizing activity.

### 2.3. Statistical Analysis

Categorical variables were compared between patients with unvaccinated and vaccinated patients using the Chi-squared and Fisher’s exact tests, while continuous variables were compared using the Mann–Whitney U test after the Shapiro–Wilk normality test.

To analyze the neutralizing activity based on vaccination status, the categorical variable (sVNT positivity) and continuous variable (sVNT % inhibition) between different vaccination statuses were compared using previously described methods. To assess cross-neutralizing activity among patients infected with various SARS-CoV-2 variants, the neutralizing activity of each variant infection across the different variants was examined using the Friedman test with Dunn’s correction. The statistical significance of the neutralizing activity for samples infected with each variant, stratified by unvaccinated and vaccinated groups, was determined using the Mann–Whitney U test.

The correlation between neutralizing activity (sVNT % inhibition) and RT-PCR Ct values was also assessed. The correlation between the Ct values of patients infected with delta, BA.1, or BA.2 and sVNT signal inhibition (%) against the corresponding variant type was analyzed using the Spearman correlation test, according to vaccination status. Statistical analyses were performed using SPSS for Windows v26.0 (IBM, Armonk, NY, USA) and GraphPad Prism version 10.2.0 for Windows (GraphPad Software, Boston, MA, USA).

### 2.4. Ethics Statement

The study protocol was reviewed and approved by the Institutional Review Board (IRB) of the Korea University Anam Hospital (protocol code 2022AN0581; 26 December 2022). The IRB granted a waiver of informed consent due to the retrospective nature of the study and the use of remnant and de-identified samples.

## 3. Results

### 3.1. Baseline Characteristics

The characteristics of the 97 patients with COVID-19 according to the vaccination status are shown in Table 1. The median time elapsed from symptom onset to admission was one day. The median age was 73 years, and all patients were Korean, with the majority being male (*n* = 52, 53.6%). CCI scores indicated that 57 patients (58.8%) had underlying medical conditions. Sixteen patients (16.5%) were immunocompromised. Of the population, 45 (46.4%) were unvaccinated. Baseline characteristics were not significantly different between the two groups, except for CCI.

### 3.2. SARS-CoV-2 Variant Identification

Out of the total patients, 51 (52.6%) were infected with the SARS-CoV-2 delta variant, and 46 (47.4%) were infected with the SARS-CoV-2 omicron variant, comprising 37 (38.1%) with the omicron sublineage BA.1 and 9 (9.3%) with BA.2 (Table 1).

### 3.3. Neutralizing Activity between Unvaccinated and Vaccinated Patients and against Different SARS-CoV-2 Variants

**Figure 1:**
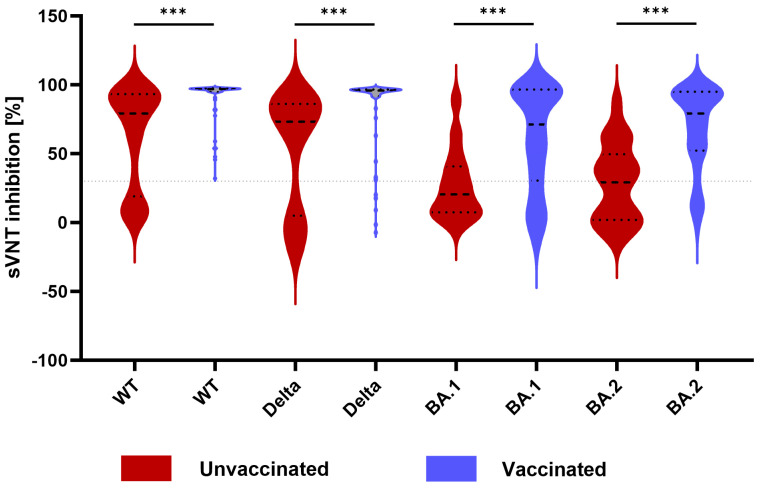
Violin charts of sVNT inhibition against different SARS-CoV-2 variants, according to vaccination status. Neutralizing activity against WT, delta, BA.1, and BA.2 is shown for all patients stratified into unvaccinated (red) and vaccinated (blue) groups. The dashed and dotted lines inside the violin plots represent the median and quartiles, respectively. The dotted line outside the plots represents the cut-off for positivity of the assay (30% signal inhibition). The width of each curve corresponds to the approximate distribution of patients in each region. Statistical significance between vaccination status is shown by the Mann–Whitney U test. *** *p* < 0.001. Abbreviations: sVNT, surrogate virus neutralization test; WT, wild-type.

To assess how vaccination affects neutralizing activity, sVNT results were compared between unvaccinated and vaccinated patients. The positivity and median % inhibition of neutralizing activity against the WT, delta, and omicron variants (BA.1 and BA.2) were significantly higher in the vaccinated group than those in the unvaccinated group (Table 1 and Figure 1). The distribution of variants was not associated with vaccination status (Table 1).

**Figure 2:**
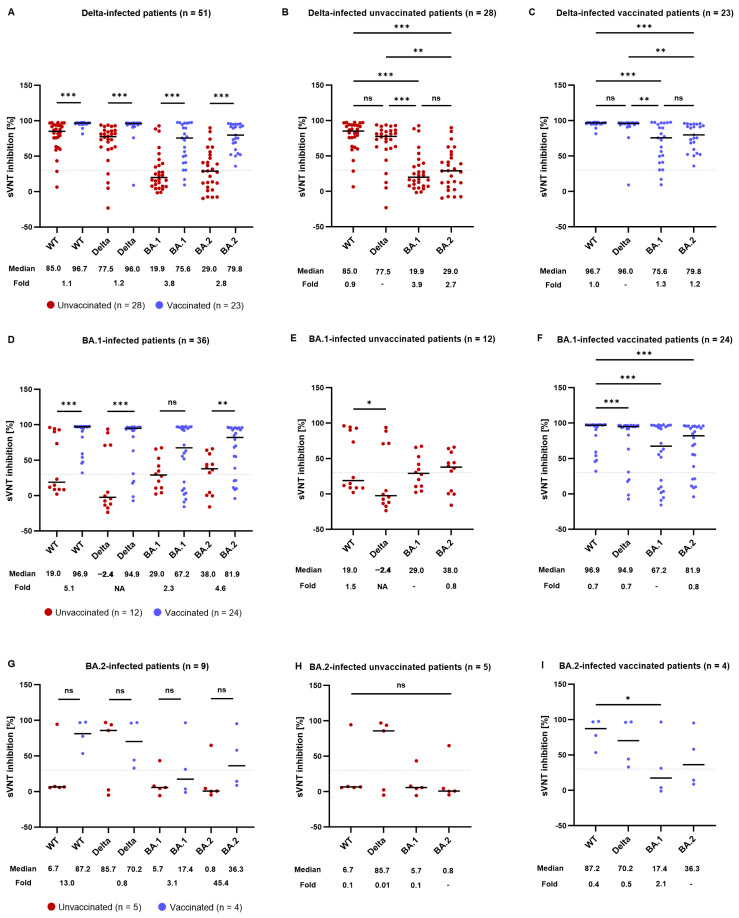
Neutralizing activity in delta or omicron (BA.1 or BA.2)-infected patients against SARS-CoV-2 WT and variants, according to vaccination status. (**A**) The neutralizing activity (sVNT % inhibition) of delta-infected serum against WT, delta, omicron BA.1, and omicron BA.2 is shown for unvaccinated patients (red) or vaccinated patients (blue). (**B**,**C**) Lines show neutralizing activity median for delta-infected (**B**) unvaccinated or (**C**) vaccinated patients. (**D**) The neutralizing activity (sVNT % inhibition) of BA1-infected serum against WT, delta, omicron BA.1, and omicron BA.2 is shown for unvaccinated patients (red) or vaccinated patients (blue). (**E**,**F**) Lines show neutralizing activity median for BA1-infected (**E**) unvaccinated or (**F**) vaccinated patients. (**G**) The neutralizing activity (sVNT % inhibition) of BA.2-infected serum against WT, delta, omicron BA.1, and omicron BA.2 is shown for unvaccinated patients (red) or vaccinated patients (blue). (**H**,**I**) Lines show neutralizing activity median for BA.2-infected (**H**) unvaccinated or (**I**) vaccinated patients. The line indicates the median, which is also represented below the plot with a fold decrease for other variants relative to vaccinated patients (**A**,**D**,**G**), delta (**B**,**C**), BA.1 (**E**,**F**), or BA.2 (**H**,**I**). Dotted lines indicate the limit of positivity (30% signal inhibition). Statistical significance between unvaccinated and vaccinated samples was determined using the Mann–Whitney U test. Statistical significance across variants was determined using the Friedman test with Dunn’s correction. * *p* < 0.05, ** *p* < 0.01, *** *p* < 0.001, and ns = non-significant. Abbreviations: sVNT, surrogate virus neutralization test; WT, wild-type; NA, not applicable.

To examine the impact of vaccination on the neutralizing activity in individuals infected with different SARS-CoV-2 variants, patients were categorized based on their vaccination status and the specific variant they had contracted (Table 2 and Figure 2). In the patients infected with the delta variant, sVNT positivity against BA.1 and BA.2 was significantly higher in the vaccinated patients, compared to that in the unvaccinated patients (Table 2). The median % inhibition against every variant and the WT was significantly higher in the vaccinated patients than in the unvaccinated patients (Figure 2 and Table 2), indicating that vaccination prior to infection increased the neutralizing activity.

In the unvaccinated patients infected with the delta variant, the median % signal inhibition against the delta variant was the highest among the variants (Figure 2 and Table 2). We also observed statistically significant decreases in binding to BA.1 and BA.2 variants (3.9- and 2.7-fold, respectively) (Figure 2). Binding to the delta variants was higher in previously vaccinated individuals who experienced breakthrough delta variant infection than in unvaccinated individuals, suggesting higher levels of neutralizing activity against delta variant reinfection. Furthermore, antibodies from these vaccinated individuals exhibited a smaller fold decrease in binding to BA.1 and BA.2 variants, indicating high cross-neutralizing activity against omicron variants (Figure 2).

The positivity rate against the WT and each variant was higher in the vaccinated patients infected with the BA.1 variant than in the unvaccinated patients (Table 2). Moreover, the median inhibition levels against the WT, delta, and BA.2 variants were significantly higher in the vaccinated patients than those in the unvaccinated patients (Figure 2 and Table 2). The decrease in the median titer was not significant in the unvaccinated patients. In the vaccinated patients who experienced breakthrough BA.1 infection, BA.1-triggered antibodies were fairly cross-reactive for all tested variants, with fold decreases below one (Figure 2).

Among the patients infected with the BA.2 variant, the vaccinated patients showed a higher sVNT positivity rate against all the strains tested than the unvaccinated patients (Table 2). In addition, the vaccinated patients revealed a greater median % inhibition against the BA.1 and BA.2 variants (Figure 2 and Table 2).

Given the higher pathogenicity of the delta variant in comparison to the omicron variants [10], it is valuable to assess the impact of the delta variant on neutralizing activity relative to that of the omicron variants. In unvaccinated patients, the sVNT positivity against both WT and delta variant was greater among those infected with the delta variant compared to those infected with the omicron variants (Table S1). This pattern is similarly reflected in the median % signal inhibition (Figure S1). In the case of vaccinated patients, the sVNT positivity against the BA.1 and BA.2 variants was higher among the delta-infected patients, as opposed to those infected with the omicron variants (Table S1). No statistically significant difference was observed in the median % inhibition against variants (Figure S1).

The Ct values were also assessed based on each variant, which were not significantly different between unvaccinated and vaccinated patients (Table 2).

### 3.4. Correlation between Ct Value and Neutralizing Activity

**Figure 3:**
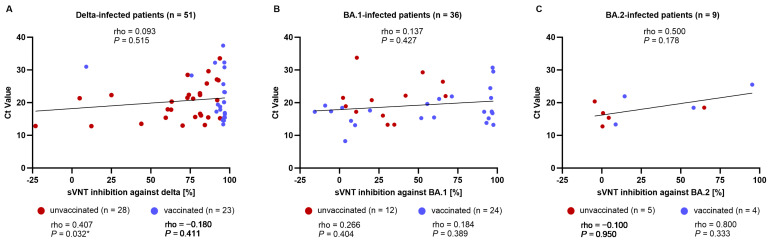
Correlation between Ct value and neutralizing activity according to variant type and respective sVNT. Correlation plots show association between Ct value in (**A**) delta-infected patients and sVNT inhibition against delta, (**B**) BA.1-infected patients and sVNT inhibition against BA.1, and (**C**) BA.2-infected patients and sVNT inhibition against BA.2. Non-parametric Spearman correlation coefficient (rho) with *p* values is shown above the graph for all patients, including both unvaccinated (red) and vaccinated patients (blue), for which linear regression is depicted as solid lines. Rho and *p* values are also represented below the plot according to vaccination status. * *p* < 0.05. Abbreviations: Ct, cycle threshold; sVNT, surrogate virus neutralization test.

Next, we compared the correlation between neutralizing activity and Ct values according to vaccination status (Figure 3). The Ct values of patients infected with a specific variant (delta, BA.1, or BA.2) were compared with sVNT inhibition against the respective variants. In the unvaccinated individuals infected with the delta variant, there was a significant correlation between neutralizing activity and Ct values (rho = 0.407, *p* = 0.032). However, this relationship was not observed in the vaccinated individuals. No significant correlations were noted for patients infected with BA.1 or BA.2 variants.

## 4. Discussion

There are still many unknowns regarding the immune response to COVID-19, even with the efforts of the scientific community and the immunization of a sizable portion of the global population [1]. An intriguing aspect of our study is the examination of the neutralizing activity of delta and omicron infections against past and subsequent SARS-CoV-2 variants, stratified by vaccination status. Monitoring the serological response to SARS-CoV-2 infection and vaccination is crucial for estimating the neutralizing efficacy and informing vaccination policies. The constant introduction of novel viral variants [15] makes it imperative to evaluate the degree to which prior immunity acquired through vaccination or natural infection protects against newly circulating strains [16]. In this study, the neutralizing activity against both BA.1 and BA.2 variants decreased substantially in unvaccinated individuals infected with the delta variant. In contrast, delta breakthrough infections were associated with enhanced omicron BA.1 and BA.2 cross-neutralization, suggesting the adaptive capacity of the immune response in vaccinated individuals. In patients infected with omicron BA.1, vaccination induced strong neutralizing activity against the WT, delta, and omicron BA.2 variants. Neutralizing activity in the patients infected with the BA.2 variant showed a similar pattern without statistical significance, mainly due to the small sample size (n = 9). With the emergence of new variants, as exemplified by the omicron variant, there is a crucial need for epidemiological studies [12]. These studies are necessary for understanding not only the protection offered through vaccination, but also past infection [12]. In this investigation, the limited neutralizing antibody responses after infection in unvaccinated individuals indicated that relying solely on convalescent immunity without vaccination is unlikely to offer adequate protection against emerging variants. Our results are corroborated by the recent studies that delta and omicron breakthrough infections greatly increased the neutralizing activity against different variants [8,17,18]. These findings are especially significant, since monitoring infections and variant emergence will remain a crucial feature of managing present and future COVID-19 transmission [12]. In individuals who have been vaccinated, the occurrence of breakthrough infections is not uncommon. The primary objective of vaccination has been to provide protection against severe disease rather than completely preventing transmission [8]. A meta-analysis showed that a significant association was observed between vaccination history and a substantial reduction in the risk of both reinfection and severe COVID-19 [19]. Additionally, an earlier study had shown that the SARS-CoV-2 virus mutates during the infection phase [20], and hosts who have not received vaccinations may serve as reservoirs for novel variants of concern [21]. In our study, the enhanced neutralizing activity through vaccination reinforces the importance of scheduled vaccinations, even for individuals with a history of infection, particularly if they were previously unvaccinated.

The omicron variant has been associated with milder disease outcomes, including a reduced risk of hospitalization and death compared to prior lineages such as the delta variant [10,22]. Previous data indicate that omicron breakthrough infections lead to a slower rise in neutralizing antibodies and lower antibody levels than delta infections [10,23]. This observation is supported by our current study, which demonstrates higher neutralizing activity in the serum of delta-infected patients compared to omicron-infected patients. Our findings help to explain the widespread infections caused by the immune-evasive omicron globally, affecting both vaccinated and unvaccinated individuals.

This study also assessed the correlation between neutralizing antibody responses and a viral load proxy (Ct value). After infection, neutralizing antibodies rise rapidly and are essential for both viral clearance and viral illness prevention [24]. Neutralizing activity has previously been inversely linked to viral load in COVID-19 among unvaccinated individuals [25]. In our study, this correlation remains consistent in unvaccinated patients infected with the delta variant.

While our study focuses on the immune response to evolving SARS-CoV-2 variants and the implications for vaccination strategies during the COVID-19 pandemic, we recognize the importance of considering broader implications for response efforts beyond the COVID-19 pandemic. As COVID-19 transitions from epidemic to endemic state [26], it is anticipated to persist for an extended period. Moreover, we are just beginning to systematically assess previous measures to enhance our preparedness for future pandemics [26]. Our findings demonstrate the potential of breakthrough infections to enhance cross-neutralizing activity against different variants, providing valuable insights into the dynamics of immunity and breakthrough infection management strategies in the endemic phase. In addition, preparedness for the next pandemic requires a multidisciplinary approach that integrates insights from host, pathogen, and environmental factors. By elucidating the impact of vaccination on immune responses to SARS-CoV-2 variants, our research offers significant understanding into the interplay between viral evolution, host immunity, and vaccination strategies, which are applicable to a wide range of infectious diseases with pandemic potential.

Various factors influence the neutralizing activity of SARS-CoV-2, including the method of acquiring the immunity (via infection or vaccination) with different types of vaccines [27], viral antigenic variation [28], and timing between repeat exposures such as vaccine doses and vaccination-to-infection intervals [29,30]. Additionally, host variables such as age, sex, comorbidities, and medications also contribute to the neutralizing activity [27]. Therefore, it is important to consider these factors when evaluating the limitations of this study. For example, the heterogeneity in vaccination history among participants, with four different vaccination types, may lead to varying impacts of vaccine-induced immunity across different types. Additionally, variation in the interval between the last vaccination and infection among vaccinated patients could also influence the outcomes of this study. Furthermore, the presence of immunocompromised participants, comprising 16.5% of the study population, may affect the host antibody response against SARS-CoV-2 variants, complicating the interpretation of results. In addition, the analysis of serum samples was conducted at a single time point to assess neutralizing activity, preventing an exploration of the temporal dynamics of neutralizing immunity. Future studies should explore the consequences of evolving immunity post-SARS-CoV-2 infection. Moreover, we did not assess neutralizing activity using the gold standard Plaque Reduction Neutralization Test (PRNT) method due to its requirement for a Biosafety Level 3 facility or the utilization of a pseudotyped virus. However, an excellent correlation was established between the PRNT and sVNT assays [31]. Finally, this study was based on a single-center, retrospective cohort consisting of 97 individuals. The sample size could potentially be a limiting factor, particularly for the analysis involving BA.2-infected patients.

Despite these limitations, our research compared the neutralizing activity between unvaccinated and vaccinated patients infected with the delta or omicron variant. It is crucial to acknowledge the growing challenge of evaluating the protection offered by breakthrough infections, particularly when comparing them to unvaccinated infected patients, given the limited number of unvaccinated individuals in many countries [8]. Our research holds substantial merit in addressing this challenge.

## 5. Conclusions

Vaccination followed by a breakthrough infection significantly increased the neutralizing activity against SARS-CoV-2 variants compared to that in unvaccinated patients infected with SARS-CoV-2. Importantly, vaccination significantly increased cross-neutralizing activity against different SARS-CoV-2 variants in delta and omicron BA.1-infected patients. The increased protection offered by breakthrough infection may provide a practical implication in optimizing vaccination strategies.

## Figures and Tables

**Table 1:** Baseline characteristics, identification of SARS-CoV-2 variants, and neutralizing activity of hospitalized patients with COVID-19, according to vaccination status.

	All Patients (*n* = 97)	Unvaccinated (*n* = 45)	Vaccinated (*n* = 51)	*p* Value
Symptom onset to hospital admission (days), median [IQR]	1 [0–3]	1 [0–3]	1 [0–4]	0.471
Age, median [IQR]	73 [65–82]	71 [58–82.5]	74 [67–81]	0.646
Female sex	45 (46.4%)	24 (53.3%)	21 (38.2%)	0.234
Male sex	52 (53.6%)	21 (46.7%)	30 (61.8%)	
Nationality				
Korean	97 (100%)			
Non-Korean	0 (0%)			
Charlson Comorbidity Index score				
0	40 (41.2%)	23 (51.1%)	15 (29.4%)	0.006 **
1–2	32 (33.0%)	17 (37.8%)	16 (31.4%)	
≥3	25 (25.8%)	5 (11.1%)	20 (39.2%)	
Immunosuppression				
Any type	16 (16.5%)	6 (13.3%)	10 (19.6%)	0.410
Cancer treatment (chemotherapy or radiation)	10 (10.3%)	4 (8.9%)	6 (11.8%)	0.746
Organ transplantation	5 (5.2%)	1 (2.2%)	4 (7.8%)	0.367
Corticosteroids	4 (4.1%)	1 (2.2%)	3 (5.9%)	0.620
HIV/AIDS	0 (0%)			
PrEP/PEP	0 (0%)			
Vaccination status				
Unvaccinated	45 (46.4%)			
Partially vaccinated ^a^	1 (1.0%)			
Fully vaccinated	51 (52.6%)			
BNT-BNT			16 (31.4%)	
ChAd-ChAd			11 (21.6%)	
ChAd-BNT			1 (2.0%)	
BNT-BNT-BNT			12 (23.5%)	
ChAd-BNT-BNT			2 (3.9%)	
ChAd-ChAd-BNT			5 (9.8%)	
ChAd-ChAd-mRNA			4 (7.8%)	
Last vaccination dose to infection (days), median [IQR]			96.5 [62.8–149.5]	
Ct value of first PCR on admission, median [IQR]	18.5 [15.5–22.6]	20.3 [15.4–22.4]	17.9 [15.5–23.2]	0.791
Symptom onset to first PCR (days), median [IQR]	2 [0–5]	2 [0–5]	1 [0–5]	0.184
SARS-CoV-2 variant				
Delta	51 (52.6%)	28 (62.2%)	23 (45.1%)	0.093
Omicron	46 (47.4%)	17 (37.8%)	28 (54.9%)	
Omicron BA.1	37 (38.1%)	12 (26.7%)	24 (47.1%)	
Omicron BA.2	9 (9.3%)	5 (11.1%)	4 (7.8%)	
Surrogate virus neutralization test Positivity (≥30% signal inhibition)				
WT	84 (86.6%)	32 (71.1%)	51 (100%)	<0.001 ***
Delta	77 (79.4%)	31 (68.9%)	46 (90.2%)	0.009 **
BA.1	55 (56.7%)	16 (35.6%)	39 (76.5%)	<0.001 ***
BA.2	65 (67.0%)	22 (48.9%]	43 (84.3%)	<0.001 ***
Neutralizing antibody titer (% signal inhibition), median [IQR]				
WT	94.3 [62.8–96.9]	79.2 [19.0–93.3]	96.8 [94.7–97.4]	<0.001 ***
Delta	88.6 [51.9–96.0]	73.3 [4.9–86.1]	95.8 [91.6–96.6]	<0.001 ***
BA.1	36.9 [9.6–83.4]	20.5 [7.3–40.8]	71.2 [30.4–96.5]	<0.001 ***
BA.2	52.2 [13.3–86.2]	29.2 [1.9–49.5]	79.1 [52.2–95.0]	<0.001 ***
Symptom onset to serologic tests (days), median [IQR]	7 [3–16.0]	6 [3–17.5]	8.5 [4.8–14.0]	0.462

Abbreviations: SARS-CoV-2, severe acute respiratory syndrome coronavirus 2; COVID-19, coronavirus disease 2019; IQR, interquartile range; HIV, human immunodeficiency virus; AIDS, acquired immunodeficiency syndrome; PrEP, pre-exposure prophylaxis; PEP, post-exposure prophylaxis; BNT, BNT162b2; ChAd, ChAdOx1 nCoV-19; mRNA, mRNA-1273; Ct, cycle threshold; PCR, polymerase chain reaction; WT, wild-type. ^a^ One partially vaccinated patient was excluded for the further surrogate virus neutralization test analysis. ** *p* < 0.01, *** *p* < 0.001.

**Table 2:** Results of the surrogate virus neutralization test in unvaccinated and vaccinated patients infected with delta or omicron SARS-CoV-2.

	Delta-Infected Patients			BA.1-Infected Patients			BA.2-Infected Patients		
Surrogate Virus Neutralization Test	Unvac (*n* = 28)	Vac (*n* = 23)	*p* Value	Unvac (*n* = 12)	Vac (*n* = 24)	*p* Value	Unvac (*n* = 5)	Vac (*n* = 4)	*p* Value
Positivity (≥30% signal inhibition)									
WT	26 (92.9%)	23 (100%)	0.495	5 (41.7%)	24 (100%)	<0.001 ***	1 (20%)	4 (100%)	0.048 *
Delta	24 (85.7%)	22 (95.7%)	0.362	4 (33.3%)	20 (83.3%)	0.007 **	3 (60%)	4 (100%)	0.444
BA.1	9 (32.1%)	21 (91.3%)	<0.001 ***	7 (58.3%)	16 (66.7%)	0.720	1 (20%)	2 (50%)	0.524
BA.2	13 (46.4%)	23 (100%)	<0.001 ***	8 (66.7%)	18 (75.0%)	0.700	1 (20%)	2 (50%)	0.524
Neutralizing antibody titer (% signal inhibition), median [IQR]									
WT	85.0 [66.5–93.6]	96.7 [95.3–97.2]	<0.001 ***	19.0 [8.7–92.1]	96.9 [84.4–97.4]	<0.001 ***	6.7 [5.6–50.8]	87.2 [59.5–97.3]	0.064
Delta	77.5 [61.3–86.2]	96.0 [94.0–96.6]	<0.001 ***	−2.4 [−13.0–71.1]	94.9 [68.0–96.5]	<0.001 ***	85.7 [−1.2–95.3]	70.2 [35.8–96.7]	0.730
BA.1	19.9 [7.7–39.0]	75.6 [41.1–96.6]	<0.001 ***	29.0 [10.7–50.1]	67.2 [8.0–96.4]	0.112	5.7 [−0.6–25.6]	17.4 [0.1–80.2]	0.905
BA.2	29.0 [7.7–50.7]	79.8 [59.3–94.7]	<0.001 ***	38.0 [1.4–55.3]	81.9 [25.7–95.5]	0.006 **	0.8 [−2.0–34.7]	36.3 [10.2–86.0]	0.111
SO to serum sample collection (day), median [IQR]	9 [3–19]	7 [4.8–17.8]	0.746	4 [0.3–8.0]	9 [4–12.8]	0.068	4 [1.5–12.5]	5.5 [1.3–15.8]	0.706
Ct value, median [IQR]	20.6 [15.4–22.7]	19.4 [15.6–28.3]	0.529	21.1 [16.4–25.3]	17.3 [15.2–20.8]	0.128	16.8 [14.1–19.4]	20.2 [14.6–24.6]	0.413
SO to PCR (day), median [IQR]	2 [1–7]	2.5 [0–5.0]	0.370	2.5 [0.3–7.8]	2 [0–6.5]	0.571	3 [0.5–3.0]	0 [0–1.5]	0.143

Abbreviations: SARS-CoV-2, severe acute respiratory syndrome coronavirus 2; Unvac, unvaccinated patients; Vac, vaccinated patients; WT, wild-type; IQR, interquartile range; SO, symptom onset; PCR, polymerase chain reaction. * *p* < 0.05, ** *p* < 0.01, *** *p* < 0.001.

## Data Availability

In the article or as Supplementary Information, all data pertinent to the study are included.

## Supplementary Materials

The following supporting information can be downloaded at: https://www.mdpi.com/article/10.3390/microorganisms12030509/s1, Figure S1: Neutralizing antibody titers in delta or omicron (BA.1 or BA.2)-infected patients against SARS-CoV-2 WT and variants, according to the variant contracted.; Table S1: Results of the surrogate virus neutralization test in delta or omicron SARS-CoV-2 infected patients.
