# Supplementary material for: Comparison of Neutralizing Activity between Vaccinated and Unvaccinated Hospitalized COVID-19 Patients Infected with Delta, Omicron BA.1, or Omicron BA.2 Variant
Source: Microorganisms. 2024 Mar 2;12(3):509. doi: 10.3390/microorganisms12030509 (PMC10974415; doi:10.3390/microorganisms12030509)
Supplement: Supplementary file 1 [file microorganisms-12-00509-s001.zip › microorganisms-2886516-supplementary.pdf]

Supplementary Materials

Comparison of neutralizing activity between vaccinated and unvaccinated hospitalized COVID-19 patients infected with delta, omicron BA.1, or omicron BA.2 variant

List of Supplementary Materials

Supplementary Figures

Figure S1. Neutralizing antibody titers in delta or omicron (BA.1 or BA.2)-infected patients against SARS-CoV-2 WT and variants, according to the variant contracted.

Supplementary Tables

Table S1. Results of the surrogate virus neutralization test in delta or omicron SARS-CoV-2 infected patients.

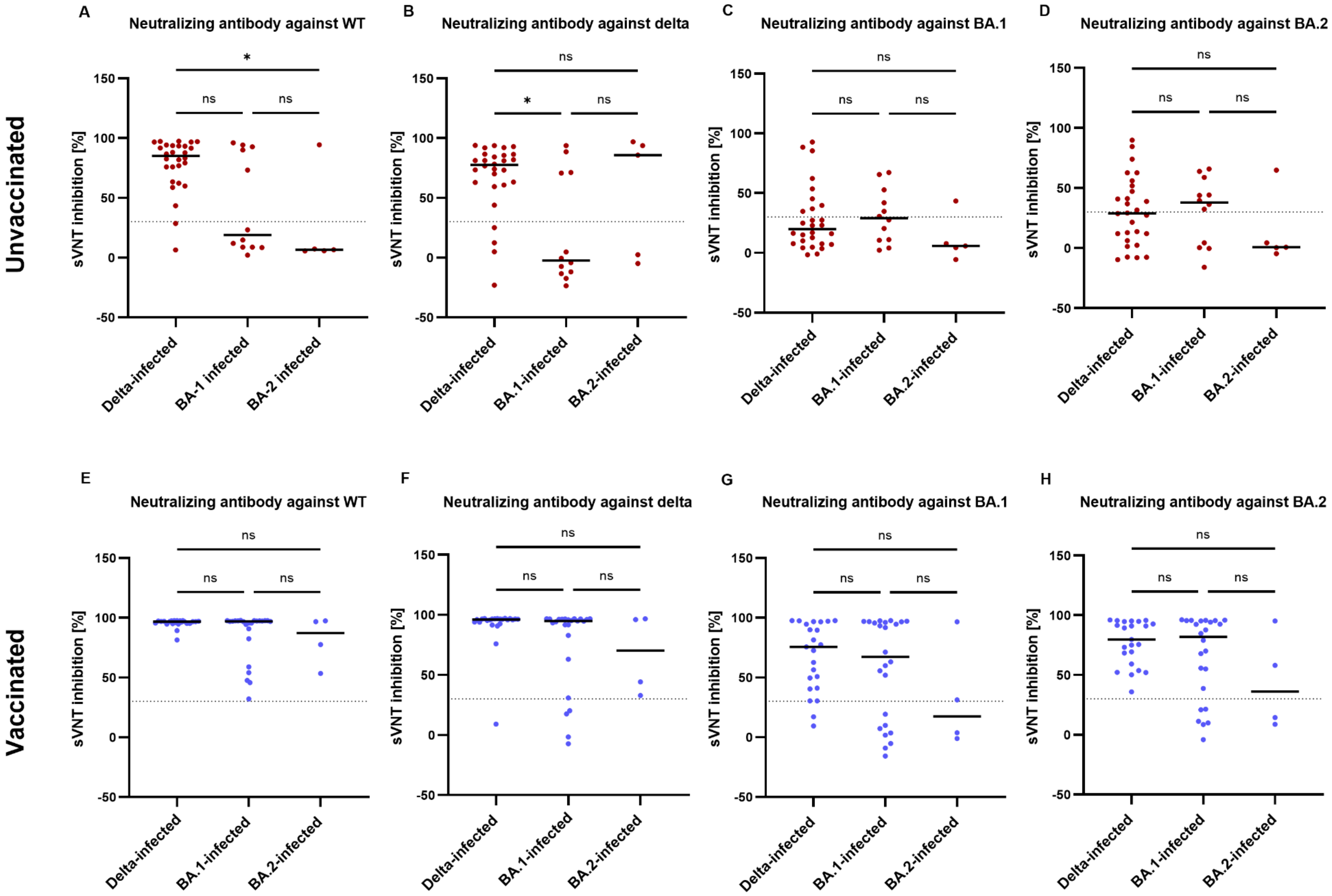
Unvaccinated
Vaccinated
A
Neutralizing antibody against WT
B
Neutralizing antibody against delta
C
Neutralizing antibody against BA.1
D
Neutralizing antibody against BA.2
E
Neutralizing antibody against WT
F
Neutralizing antibody against delta
G
Neutralizing antibody against BA.1
H
Neutralizing antibody against BA.2
sVNT inhibition [%]
150
100
50
0
-50
*
ns
Delta-infected
BA-1 infected
BA-2 infected
BA.1-infected
BA.2-infected

Figure S1. Neutralizing antibody titers in delta or omicron (BA.1 or BA.2)-infected patients against SARS-CoV-2 WT and variants, according to the variant contracted. The neutralizing antibody titers (sVNT % inhibition) against (A) WT, (B) delta, (C) omicron BA.1, and (D) omicron BA.2 are shown for unvaccinated patients (red), and against (E) WT, (F) delta, (G) omicron BA.1, and (H) omicron BA.2 for vaccinated patients (blue), categorized by the variant contracted. The lines indicate the median and dotted lines indicate the limit of positivity (30% signal inhibition). Statistical significance across the different variants was determined using the Kruskal-Wallis test with Dunn's correction. *$P < 0.05$ and ns = non-significant. Abbreviations: sVNT, surrogate virus neutralization test; WT, wild-type; NA, not applicable.

Table S1. Results of the surrogate virus neutralization test in delta or omicron SARS-CoV-2 infected patients.

Surrogate virus neutralization test	Unvaccinated patients				Vaccinated patients			
	Delta-infected (n = 28)	BA.1-infected (n = 12)	BA.2-infected (n = 5)	*P* value	Delta-infected (n = 23)	BA.1-infected (n = 24)	BA.2-infected (n = 4)	*P* value
Positivity (≥30% signal inhibition)								
WT	26 (92.9%)	5 (41.7%)	1 (20%)	<0.001	23 (100%)	24 (100%)	4 (100%)	NA
Delta	24 (85.7%)	4 (33.3%)	3 (60%)	0.004	22 (95.7%)	20 (83.3%)	4 (100%)	0.455
BA.1	9 (32.1%)	7 (58.3%)	1 (20%)	0.276	21 (91.3%)	16 (66.7%)	2 (50%)	0.045
BA.2	13 (46.4%)	8 (66.7%)	1 (20%)	0.198	23 (100%)	18 (75.0%)	2 (50%)	0.005
SO to serum sample collection (day), median [IQR]	9 [3–19]	4 [0.3–8.0]	4 [1.5–12.5]	0.141	7 [4.8–17.8]	9 [4–12.8]	5.5 [1.3–15.8]	0.628

Abbreviations: SARS-CoV-2, severe acute respiratory syndrome coronavirus 2; WT, wild-type; NA, not applicable; SO, symptom onset; IQR, interquartile range. The categorical variables were assessed using the Fisher's exact test and continuous variable was compared using the Kruskal-Wallis test.
